# Supplementary material for: A Robust Modular Automated Neuroimaging Pipeline for Model Inputs to TheVirtualBrain
Source: Front Neuroinform. 2022 Jun 14;16:883223. doi: 10.3389/fninf.2022.883223 (PMC9239912; doi:10.3389/fninf.2022.883223)
Supplement: Supplementary file 1 [file Data_Sheet_1.pdf]

Supplementary Material

Supplementary Table 1. Table of novel TVB-UKBB IDPs and their descriptions. Underlined IDP categories exist in the original UK Biobank pipeline but have been modified for the TVB-UKBB pipeline. Bolded rows define the category of IDP that the following rows fall under.

IDP name	Description
**Category: tvb_IDP_FC_distribution**[1]	**Functional connectivity summary statistics**[1]
FC_distribution_min_fMRI	Functional connectivity (Pearson correlation coefficient) minimum
FC_distribution_max_fMRI	Functional connectivity (Pearson correlation coefficient) maximum
FC_distribution_median_fMRI	Functional connectivity (Pearson correlation coefficient) median
FC_distribution_mean_fMRI	Functional connectivity (Pearson correlation coefficient) mean
FC_distribution_mean_to_max_fMRI	Functional connectivity (Pearson correlation coefficient) difference between mean and max
FC_distribution_median_to_max_fMRI	Functional connectivity (Pearson correlation coefficient) difference between median and max
FC_distribution_range_fMRI	Functional connectivity (Pearson correlation coefficient) range
FC_distribution_proportion_neg_fMRI	Functional connectivity proportion negative

FC_norm_MSE_fMRI	Functional connectivity - mean squared error for a Gaussian distribution fit
**Category: tvb_IDP_SC_distribution[1]**	**Structural connectivity summary statistics[1]**
SC_distribution_min	Structural connectivity (log of Probability of connection) minimum
SC_distribution_max	Structural connectivity (log of Probability of connection) maximum
SC_distribution_median	Structural connectivity (log of Probability of connection) median
SC_distribution_mean	Structural connectivity (log of Probability of connection) mean
SC_distribution_mean_to_max	Structural connectivity (log of Probability of connection) difference between mean and max
SC_distribution_median_to_max	Structural connectivity (log of Probability of connection) difference between median and max
SC_distribution_range	Structural connectivity (log of Probability of connection) range
SC_distribution_proportion_neg	Proportion of non-zero Structural connectivity (Probability of connection) values
SC_nan_lines	Structural connectivity – indices of ROIs with only NaN structural connectivity (Probability of connection) values
SC_num_nan	Structural connectivity - number of ROIs all NaN connections

SC_lognorm_MSE[2]	Structural connectivity - mean squared error for lognorm distribution fit[2]
**Category: tvb_IDP_FIX_classes**	**MELODIC independent component (IC) class proportions: proportion of ICs that are signal, noise, or unknown**
FIX_prop_IC_unknown	Proportion of MELODIC ICs that are classified as unknown by FIX
FIX_prop_IC_signal	Proportion of MELODIC ICs that are classified as signal by FIX
FIX_prop_IC_noise	Proportion of MELODIC ICs that are classified as noise by FIX
**Category: tvb_IDP_MCFLIRT_disp**	**MCFLIRT displacement summary statistics**
MCFLIRT_rel_disp_min_fMRI	MCFLIRT relative displacement minimum
MCFLIRT_rel_disp_max_fMRI	MCFLIRT relative displacement maximum
MCFLIRT_rel_disp_median_fMRI	MCFLIRT relative displacement median
MCFLIRT_rel_disp_mean_fMRI	MCFLIRT relative displacement mean
MCFLIRT_rel_disp_range_fMRI	MCFLIRT relative displacement range
MCFLIRT_rel_disp_proportion_gt_one_fMRI	MCFLIRT relative displacement - proportion of time units with displacement greater than 1mm
MCFLIRT_rel_disp_num_gt_one_fMRI	MCFLIRT relative displacement - number of time units with displacement greater than 1mm

MCFLIRT_abs_disp_min_fMRI	MCFLIRT absolute displacement minimum
MCFLIRT_abs_disp_max_fMRI	MCFLIRT absolute displacement maximum
MCFLIRT_abs_disp_median_fMRI	MCFLIRT absolute displacement median
MCFLIRT_abs_disp_mean_fMRI	MCFLIRT absolute displacement mean
MCFLIRT_abs_disp_range_fMRI	MCFLIRT absolute displacement range
MCFLIRT_abs_disp_proportion_gt_one_fMRI	MCFLIRT absolute displacement - proportion of time units with displacement greater than 1mm
MCFLIRT_abs_disp_num_gt_one_fMRI	MCFLIRT absolute displacement - number of time units with displacement greater than 1mm
**Category: tvb_IDP_homotopic**	**Mean Pearson correlation coefficient value for functional connectivity between homotopic ROIs.**
FC_homotopic_mean_fMRI	Homotopic functional connectivity mean
**Category: tvb_IDP_func_TSNR**	**Temporal signal-to-noise ratios in fMRI**
fMRI_TSNR	Temporal signal-to-noise ratio in the pre-processed fMRI - reciprocal of median (across brain voxels) of voxelwise mean intensity divided by voxelwise timeseries standard deviation
fMRI_cleaned_TSNR	Temporal signal-to-noise ratio in the artefact-cleaned pre-processed fMRI - reciprocal of median (across brain voxels) of voxelwise mean intensity divided by voxelwise timeseries standard deviation
fMRI_num_vol	Number of volumes (timepoints) in fMRI scan

**Category: tvb_IDP_func_susceptibility_SNR[3]**	**Temporal signal-to-noise ratios in susceptible and non-susceptible brain regions in fMRI[3]**
fMRI_non-susceptible_TSNR	Temporal signal-to-noise ratio in the minimally-processed fMRI non-susceptible regions - median (across brain voxels) of voxelwise mean intensity divided by voxelwise timeseries standard deviation
fMRI_non-susceptible_cleaned_TSNR	Temporal signal-to-noise ratio in the artefact-cleaned pre-processed fMRI non-susceptible regions - median (across brain voxels) of voxelwise mean intensity divided by voxelwise timeseries standard deviation
fMRI_susceptible_TSNR	Temporal signal-to-noise ratio in the minimally-processed fMRI susceptible regions - median (across brain voxels) of voxelwise mean intensity divided by voxelwise timeseries standard deviation
fMRI_susceptible_cleaned_TSNR	Temporal signal-to-noise ratio in the artefact-cleaned pre-processed fMRI susceptible regions - median (across brain voxels) of voxelwise mean intensity divided by voxelwise timeseries standard deviation
**Category: tvb_IDP_all_align_to_T1**	**Discrepancy between various modalities registered to T1 space and the T1 image**
T2_FLAIR_align_to_T1	Discrepancy between the T2_FLAIR brain image (linearly-aligned to the T1) and the T1 brain image
dMRI_align_to_T1	Discrepancy between the dMRI brain image (linearly-aligned to the T1) and the T1 brain image
fMRI_align_to_T1	Discrepancy between the fMRI brain image (linearly-aligned to the T1) and the T1 brain image
fMRI_fieldmap_align_to_T1	Discrepancy between the fMRI gradient echo field map brain image (linearly-aligned to the T1) and the T1 brain image

**Category: tvb_IDP_fieldmap_func_align**	**Discrepancy between the fMRI field map brain image registered to fMRI func space and the fMRI func image**
fMRI_fieldmap_func_align	Discrepancy between the fMRI gradient echo field map brain image and the fMRI image

[1] *Summary statistics for connectivity matrices (structural, functional) are calculated without the values from the main diagonal in order to exclude reflexive connectivity.*

[2] *Structural connectivity weights were fit to a lognormal distribution based on our previous observations that the heavily skewed distributions of anatomical tract tracing weights are well captured by the tractographic methodology used in the TVB-UKBB pipeline (Shen et al., 2019 Sci Data)*

[3] *Areas susceptible to fMRI dropout are defined by the user with an ROI mask image that is compatible with the user-defined parcellation (i.e., some subset of the parcellation). In our Cam-CAN usage example, this mask includes ROIs near large sinuses (orbitofrontal cortex, inferior temporal cortex, temporal pole).*

Supplementary Table 2. Table of anatomical QC Report features. QC Report Features are listed in order of appearance in the QC Report. Each QC Report Feature is presented in the QC Report in the order their described images and outputs are processed by the pipeline.

**QC Report Feature**	**Description**
T1 registration to standard	Overlay of T1 and MNI T1 standard
T2 registration to T1	Overlay of T1 and T2
T1 brain extraction	Overlay of T1 and T1 brain mask
T1 WM segmentation	Overlay of T1 and WM
T1 GM segmentation	Overlay of T1 and GM
Unlabelled subcortex	Zoomed-in overlay of T1 and T1 brain mask
Labelled subcortex	Zoomed-in overlay of T1 and GM ROI parcellations
Labelled cortex	Overlay of T1 and GM ROI parcellations.
T2 registration	Overlay of T2 and T1
T2 FLAIR BIANCA	Overlay of T2 FLAIR lesions and T2

Supplementary Table 3. Table of Diffusion QC Report features. QC Report Features are listed in order of appearance in the QC Report. Each QC Report Feature is presented in the QC Report in the order their described images and outputs are processed by the pipeline. EDDY QUAD and EDDY SQUAD reports are also included in the TVB-UKBB QC Report.

QC Report Feature	Description
DTI Extraction	Overlay of B0 and brain mask
DTI Tensor Orientation	Overlay of DTI fractional anisotropy (FA) and DTI V1 principal diffusion tensors
DTI Registration	Overlay of T1 and DTI warped to T1 space
SynB0 Warping	Overlay of DWI_B0 and b0_u
Tractography	Overlay of DTI FA and exclude, WMGM interface, or labelled WMGM interface

Supplemental Table 4. Table of SC/FC QC Report features. QC Report Features are listed in order of appearance in the QC Report. Each QC Report Feature is presented in the QC Report in the order their described images and outputs are processed by the pipeline.

QC Report Feature	Description
Structural Connectivity	Heatmap matrix of structural connectivity (probability of connection or 'weights'). Accompanied by histogram of structural connectivity values
Functional Connectivity	Heatmap matrix of functional connectivity (Pearson correlation coefficients). Accompanied by histogram of functional connectivity values
ROI Carpet Plot	Carpet plot of resting-state fMRI time series for all ROIs
Tract Lengths	Heatmap matrix of tract lengths ('distance'). Accompanied by histogram of tract length values
rfMRI plots	MCFLIRT rfMRI estimated motion graph
tfMRI plots	MCFLIRT tfMRI estimated motion graph

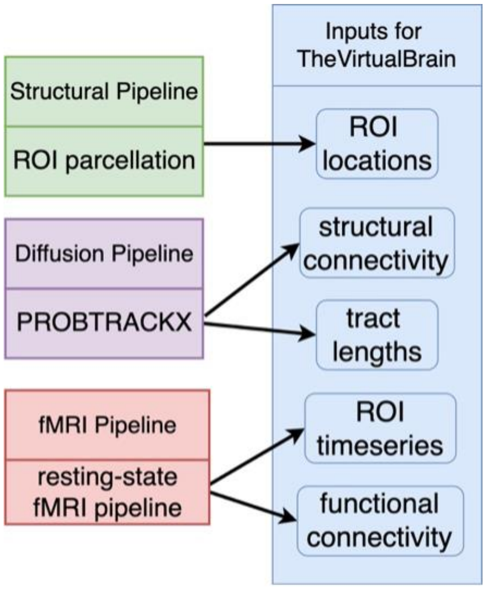

Supplementary Figure 1. Flow of outputs from the TVB-UKBB pipeline to the inputs used with TheVirtualBrain. Model inputs are generated by the pipeline and are compressed into a .zip file that is easily readable by TheVirtualBrain.

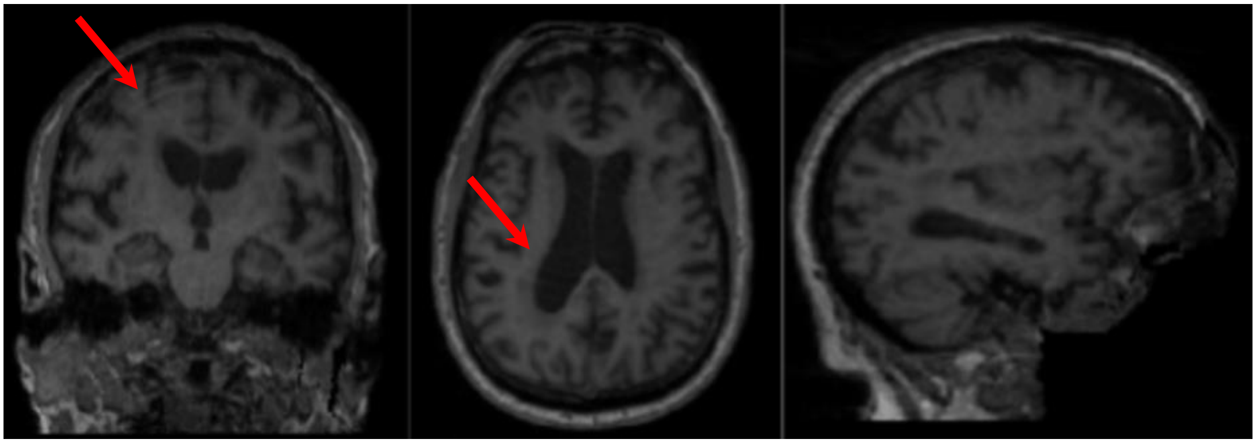

Supplementary Figure 2. T1 image analysis from the QC Report. Image shows a coronal, axial, and sagittal view of a subject T1 image (grayscale). Motion artifacts and atrophy are evident (red arrows).

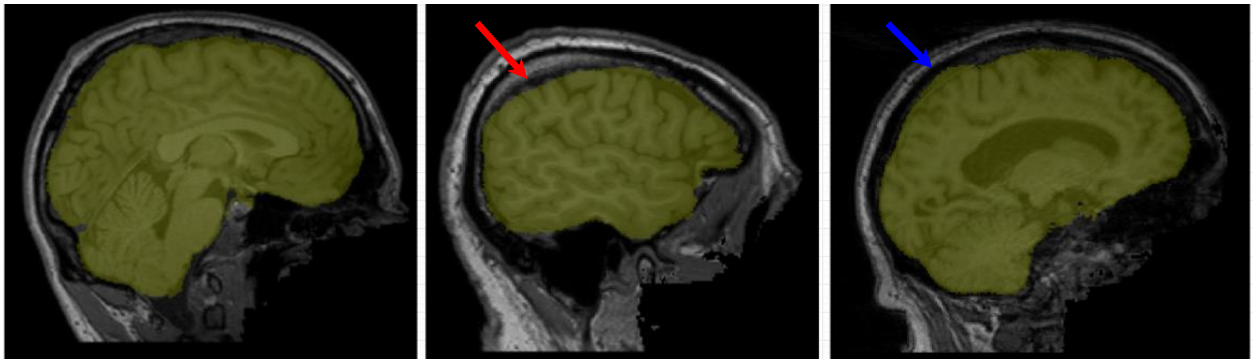

Supplementary Figure 3. T1 brain extraction analysis from the QC Report. Images show a sagittal view of a subject's brain mask (yellow) overlaid on top of their T1w image (grayscale). A slice from a well-processed subject can be seen on the left. A slice from a subject with insufficient brain inclusion in the mask can be seen in the middle (red arrow). A slice from a subject with excess dura mater inclusion in the mask can be seen on the right (light blue arrow).

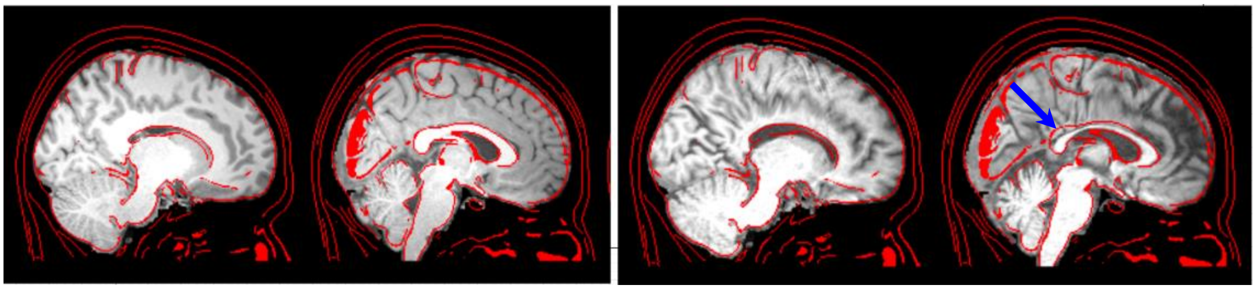

Supplementary Figure 4. T1 registration analysis from the QC Report. Images show a sagittal view of the MNI standard (red) overlaid on top of a subject's T1w image (grayscale). Two slices from a well-processed subject can be seen on the left and two slices from a poorly-processed subject can be seen on the right. The arrow (blue) points to an instance of poor alignment.

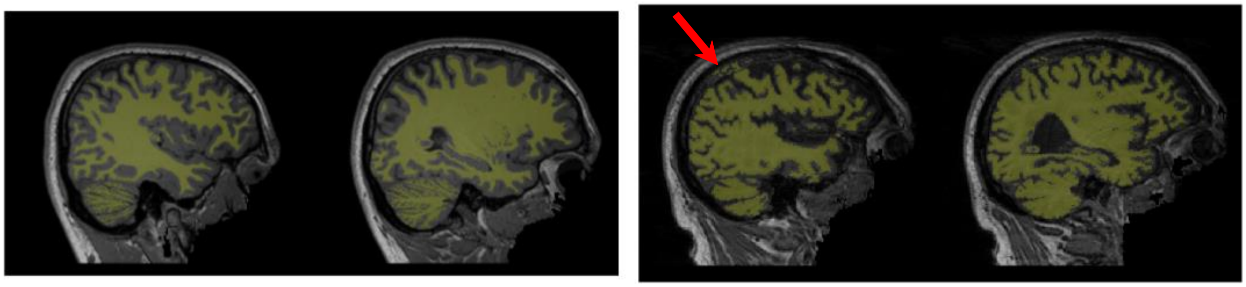

Supplementary Figure 5. T1 White Matter Segmentation from the QC Report. Images show a sagittal view of a subject's white matter mask (yellow) overlaid on top of their T1w image (grayscale). Two slices from a well-processed subject can be seen on the left. Two slices from a subject with misclassified white matter from the dura can be seen on the right (red arrow).

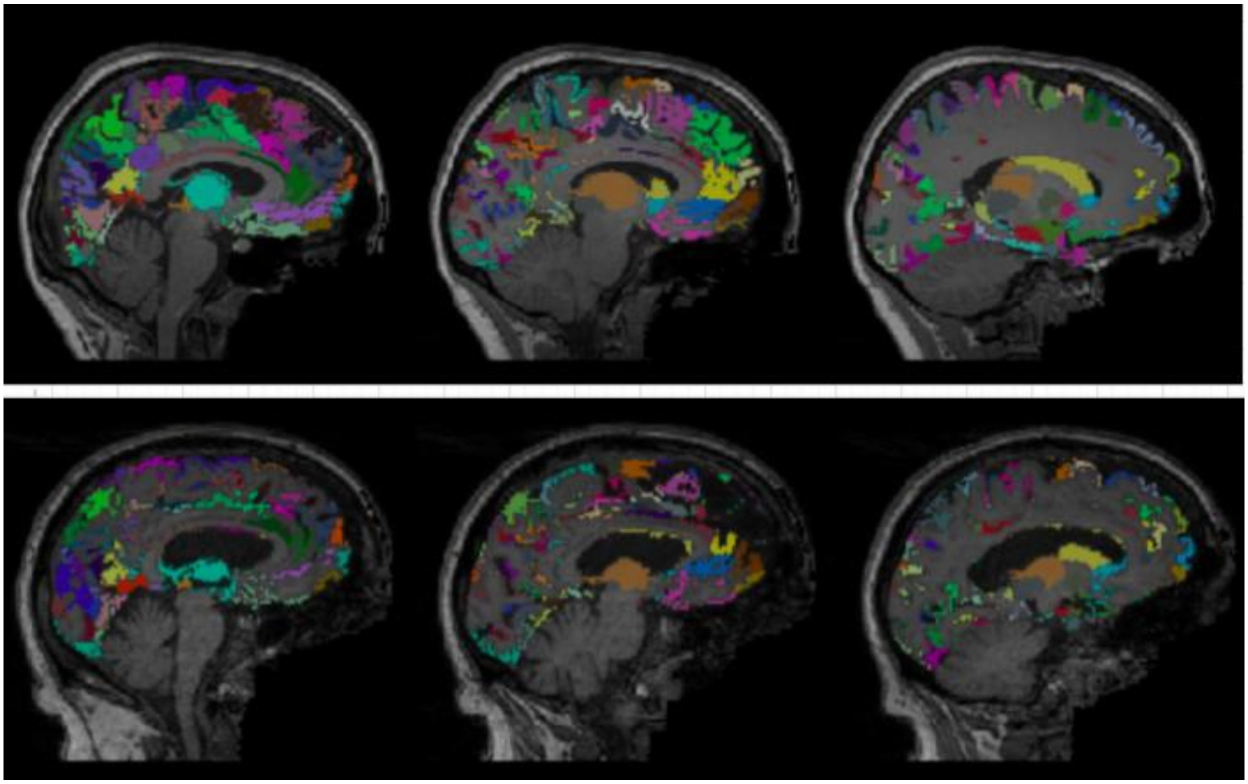

Supplementary Figure 6. T1 ROI segmentation analysis from the QC Report. Images show a view of a subject's labelled grey matter ROIs (coloured) overlaid on top of their T1w image (grayscale). Three slices from a well-processed subject can be seen on top and three slices from a poorly-processed subject, where grey matter labelling is sparse, can be seen on bottom.

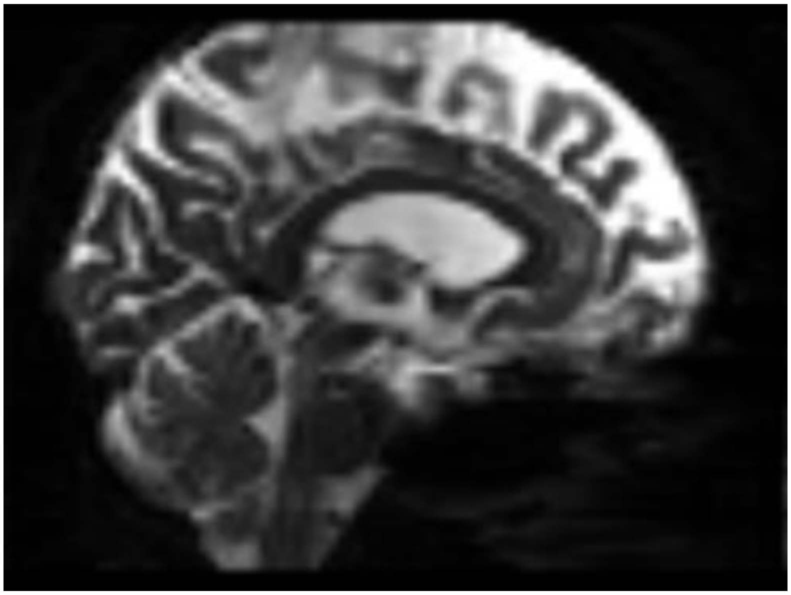

Supplementary Figure 7. dMRI B0 image analysis from the QC Report. Image shows a sagittal view of a subject's dMRI B0 image. The superior margins of the brain were outside the field of view.

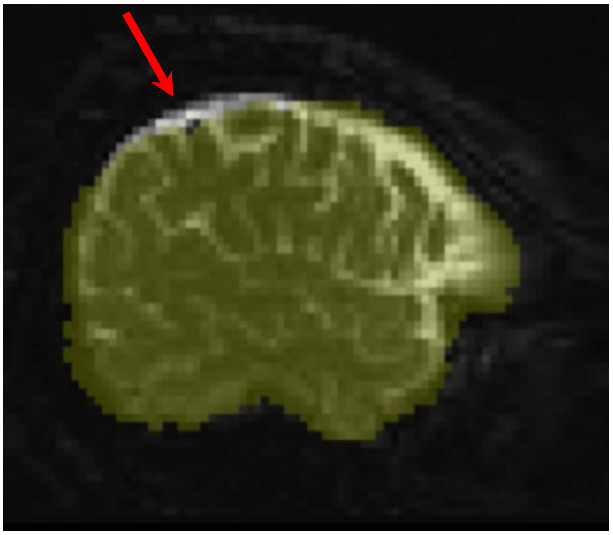

Supplementary Figure 8. dMRI brain extraction analysis from the QC Report. Image shows a sagittal view of a subject's dMRI brain mask (yellow) overlaid on top of their B0 image (grayscale). Insufficient brain inclusion in the mask excludes cortex (red arrow).

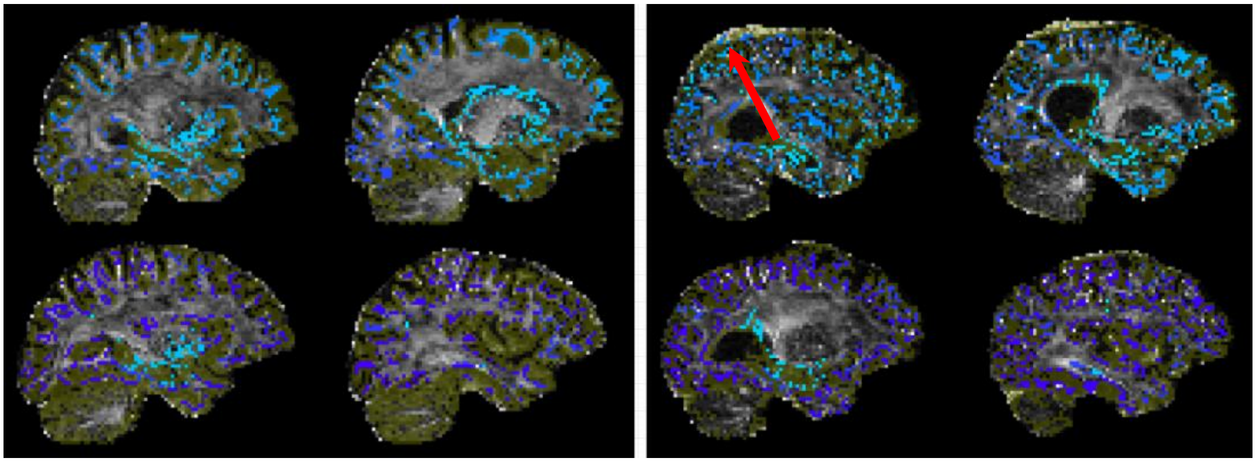

Supplementary Figure 9. dMRI tractography analysis from the QC Report. Image shows a sagittal view of a subject's tractography seeds (blue) and exclude mask (yellow) overlaid on top of their FA image. Slices from a well-processed subject (left) have seeds localized within the border of the brain. Slices from a poorly-processed subject (right) show seeds in the dura (red arrow).
